# Estimation of Life Expectancy for Dependent Population in a Multi-State Context

**DOI:** 10.3390/ijerph182111162

**Published:** 2021-10-24

**Authors:** Irene Albarrán Lozano, Pablo J. Alonso-González, José Javier Núñez-Velázquez

**Affiliations:** 1 Statistics Department, Universidad Carlos III de Madrid, Avenida Gregorio Peces-Barba Martínez 22, Colmenarejo, 28270 Madrid, Spain; ialbarra@est-econ.uc3m.es; 2 Economics Department, Universidad de Alcalá, Plaza de la Victoria 2, Alcalá de Henares, 28802 Madrid, Spain; josej.nunez@uah.es

**Keywords:** dependent people, life expectancy, Spanish population, transition probabilities

## Abstract

Population statistics show that there was an increase in life expectancy during the last century. However, this fact hides that this increase was not equal for all groups of the population. One of the most problematic cases for measuring this increase is that of the dependent population because of the absence of specific statistics. This paper describes a methodology for calculating life expectancy using multistate models that take into account the diversity of situations considered by Spanish legislation. For this purpose, statistical information contained in the national survey on disability and dependency (EDAD 2008) is used. The results suggest that life expectancies are lower than those of the general population and that they differ according to gender and intensity of suffering from this contingency. The calculations were made considering the legal framework currently existing in Spain. This fact conditions the definition of dependent person and, therefore, the set of individuals, their characteristics, and therefore, their final results.

## 1. Introduction

The increase in life expectancy is one of the greatest achievements of the 20th century. This process, together with a significant reduction in the birth rate, especially in developed countries, led to an increasing share of the older population. In fact, populations are rapidly aging around the world. This trend looks set to continue in the coming decades. As a result, the population over 60 will grow from 700 million in 2019 to more than 2 billion in 2050. The annual growth rate of the population in this age group is expected to be 2.6% compared to 1.2% for the general population. Within this segment, the largest growth is expected among the so-called oldest-old (people over 80). In 2009, they accounted for just over 14% of the over-60s and are expected to exceed 20% by 2050, when 1 in 6 people in the world will be over the age of 65, up from 1 in 11 in 2019. So, all societies in the world are in the midst of this longevity revolution [1]. The final result is a higher overall life expectancy, and throughout most of the world, survival beyond age 65 is increasing. According to this new scenario, it is necessary to develop more complex and robust demographic measures and methods [2]. Therefore, the classical approach to construct life tables needs to be revised and improved ([3,4,5]).

Although the increase in life expectancy is positive, this process was also accompanied by an increase in the so-called frail elderly, i.e., people who, because of age-related illnesses and disabilities, need help several times a day to carry out daily activities. According to [6,7], frailty is recognized as a biological syndrome associated with multisystem declines in physiological reserve and increased vulnerability to stressors, resulting in an increased risk of adverse outcomes such as disability, hospitalization, and death.

Increasing human life expectancy does not always mean gaining years of life in good health. Therefore, given that the physical and psychological conditions of this group are worse than those of the general population, it seems appropriate to ask whether their life expectancy will be the same as that of the general population and, if not, what their life expectancy will be. In an attempt to answer this question, three possible scenarios were offered in terms of senescent morbidity [8]. The scenarios are known as compression theory [9], equilibrium theory [10], and pandemic theory ([11,12]) depending on whether the reduction in mortality occurs in the final years of life, progresses at the same rate as in the general population, or whether, on the contrary, it progresses at a slower rate than that of the population as a whole. Some authors suggest that the disabled population has a higher mortality risk than the general population ([13,14,15] or [16]). Given that dependence implies aggravated disabilities, it seems reasonable that this increase in mortality is also true for this population group.

From an empirical point of view, the lack or nonexistence of data is one of the major problems when studying this aspect. There were studies of mortality in small groups. [17] analyzed a small sample of 2813 people aged over 65 years old in the provinces of Havana and Matanzas in Cuba. The authors sought both to assess risk and to detect and identify factors that influence the onset of dependence and that could be used as predictors of mortality. Another type of study was focused on analyzing mortality in the dependent population living in specialized centers. [18] studied 8902 elderly adults in 140 Japanese nursing homes. [19] conducted a similar study for South Korea, while [20] studied the mortality of dependents in Portugal, differentiating between the type of institution in charge of their care.

The use of multistate models is another possible approach to address the problem of dependency. The use of this methodology applied to health models is not new ([21,22,23,24] or [25] amongst others). From an actuarial point of view, the first theoretical work using this approach was carried out by [26]. Olivieri and Ferri [27] used a three-state stand-alone model without improvement in personal situation. Practical applications of this approach can be found in [28] or [29]. In the first case, a study is conducted on life expectancy in long-term care centers in Finland where the key variable is marital status. In the second case, the study is conducted using data from the Kerala State in India.

All of the above papers use small samples and focus on small geographic areas. Some others epidemiological studies are focused on a specific group of people (i.e., [17]) or people diagnosed with a concrete illness or disability (Alzheimer’s dementia, Parkinson, cognitive dysfunction or functional disability such as, for instance, [30,31] or [32].

An exception is [28], which uses a sample of 300,000 people in Finland. In all the works indicated, three situations are considered: healthy, unhealthy, and death. In other words, no discrimination is made between the different scenarios that can occur within the unhealthy state, derived from a greater or lesser intensity of dependence. This extreme simplification ignores the fact that some of the systems in force in Europe do take into account these different intensities. For example, the French system considers six levels, while the German system is divided into three and the Spanish system into four, three of them entitled to receive public benefits [33].

This paper aims to estimate the life expectancy of the Spanish dependent population using the multistate approach. It is not intended to estimate dependency-free life expectancies, in a similar way as [34] do for illnesses, but the objective is the estimation of total life expectancy. Five different situations are considered, linked to the different degrees of dependency with or without entitlement to public benefits plus death. In the case of dependents entitled to public benefits, the three cases included in Spanish legislation were considered. The data used were taken from the EDAD 2008 survey conducted by the Spanish National Statistics Institute (INE by its Spanish acronym). This is the last survey of this type to be carried out in Spain. Given that there is no statistical information available on mortality of dependents, the approach linked to the pandemic theory is followed. That is to say, different scenarios are assumed that relate the probability of death of the dependent population to that of the general population. Estimates were made of the life expectancy of the dependent population, distinguishing by grade, scenario, sex, and age. The life table most similar to those we are trying to obtain is the one used by the Spanish Social Security for people receiving a permanent disability pension. The life tables for each of the four analyzed situations are estimated using the the information included in the Social Security tables, the structure and mortality of the general population and the information included in EDAD 2008. The application of the multistate approach allows us to estimate the transition matrices for each age from which the life expectancies sought are obtained.

The rest of the paper is structured as follows. Section 2 gives a brief overview of the legal situation of dependency in Spain. Section 3 focuses on the description of the database used. Section 4 shows in detail the process followed for the estimation of life expectancy. Section 5 shows the results obtained. Finally, Section 6 discusses the results obtained in the paper and includes possible future lines of research related to the work carried out.

## 2. The Dependency in Spain

The Act 39/2006, on Promotion of Personal Autonomy and Care for Dependent Persons, was passed in the final days of 2006 and became effective in 1 January 2007. Dependency care is included as the fourth pillar of the welfare state. The Spanish system is based on three main principles: public and universal nature of benefits, access to benefits under conditions of equality and nondiscrimination, and the participation of all public administrations. Unlike other systems such as the French one, there is no minimum age to be eligible for the system. It is managed by the System for Autonomy and Care for Dependency (SAAD by its Spanish acronym). It is the body that regulates the basic conditions for the promotion of personal autonomy and care for people who suffer from this contingency. It is also responsible for the set of benefits and services that guarantee their care. Dependency Act guarantees a right to long-term care services to all those assessed to require care, subject to an income and asset test. The system is implemented incrementally starting with provisions for those with the severest (degree III) disability from January 2007, with the aim of covering those with milder disabilities by the end of 2014.

Dependency is assessed by means of a scale regulated by Royal Decree 504/2007. As a result, each applicant receives a score between 0 and 100 points for each indicator that in sum correspond to one of the three dependency levels. Only those with a scale of at least 25 points are eligible for public aids. Depending on the score received, the Spanish system establishes the following three degrees of dependency (article 26 of the Act39/2009):

- Degree I. Moderate dependency: a person who needs help to perform various basic activities of daily living, at least once a day or periodically and/or limited support for his/her personal autonomy. People who were classified within this grade must have a score between 25 and 49.99 points.
- Degree II. Severe dependency: a person who needs help to perform various basic activities of daily living two or three times a day, but he/she does not need/want the permanent help from a caregiver or when he/she needs extensive support services for his/her personal autonomy. People who were classified within this grade must have a score between 50 and 74.99 points.
- Degree III. Major dependency: a person who needs help with various basic activities of daily living several times a day and/or needs continued assistance from another person due to his/her total loss of physical, mental, intellectual, or sensorial autonomy. People who were classified within this grade must have a score between 75 and 100 points.

It is therefore possible to receive a score below 25 points. In that case, he or she will be a dependent person without entitlement to public benefits. These are the individuals who are included in this paper in degree 0. Once an individual is classified as a dependent with entitlement to benefits, Spanish legislation guarantees access to a range of services regardless of the autonomous region in which he or she resides, or his/her income or assets.

## 3. Data

Several statistical sources were used to carry out the proposed research. Specifically, the data used come from:

- Spanish Social Security life table for retired persons who receive a permanent annuity for disability. They were published in the State Official Gazette of 28 December 2005. This table provides information on the entire population with permanent disability, whether or not they are dependents. This table is elaborated for ages between 16 and 108 years old, both included. From now on, they are identified as $q_{x}^{SS}$.
- Life table for the general population. The PERM/F table for the year 2000 was used for this purpose, with particular reference to the year 2008. There is a life table for men and another for women. However, there is not a table for the whole population.
- Spanish population structure by age and sex in 2008. This information was taken from the INE.
- National Survey on Disability, Personal Autonomy, and Dependency (EDAD database: Encuesta sobre, Discapacidad, Autonomía personal y Situaciones de Dependencia), conducted by the Spanish National Institute of Statistics (INE) in 2008 in collaboration with the IMSERSO (Institute for the Elderly and Social Services) and the ONCE (Spanish National Organisation for Blind people). Two-stage, stratified sampling proportional to the size of each Autonomous Community was used. It collects information from more than 260,000 people living at home plus 11,000 in residential homes. Each of the 271,000 participants in the survey represents a certain number of people. This number is captured in what is called the elevation factor, so that the sum of all the factors equals the total Spanish population at the time of the survey. The interviews were carried out both with the people affected and with their relatives and/or carers. Self-perceived disability is the core concept of EDAD. It is captured by means of a set of questions that allow the assessment of possible difficulties in performing activities of daily living (hereafter ADLs). EDAD is one of the largest national survey studies on the topics of health, disability, and care in Europe. Although the name of EDAD 2008 refers to Dependency, the registers included therein only collect information on all of the disabilities included in the ICF 2001 classification (International Classification of Functioning, Disability and Health). The change from data referring to disability to other data referring to dependency was carried out considering the requirements of the current legislation in Spain. An explanation of the process followed can be found in [35].

## 4. Steps in the Estimation Process

Once the statistical sources used were described, this section is devoted to the description of the estimation process to obtain life expectancies by age, sex, and degree of dependency. Four steps were followed:

Step 1: Inclusion of sex in the Social Security life tables.

**Figure 1:**
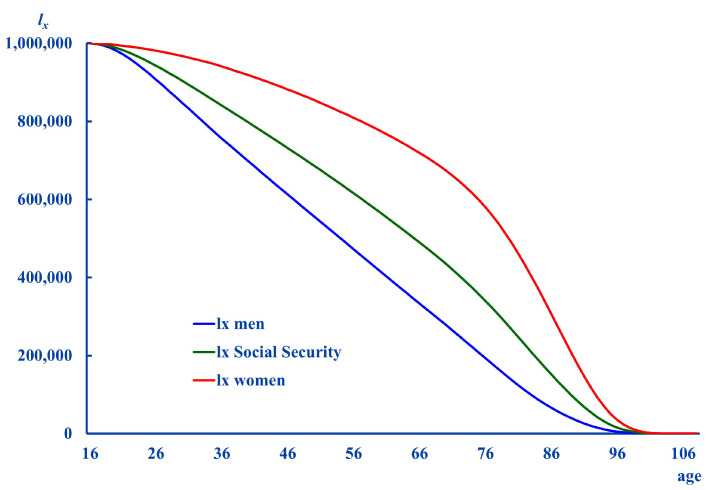
Social Security life tables considering sex.

As it was indicated in the previous section, the Social Security permanent disability tables are those used by the Spanish public administration to evaluate the life expectancy of individuals with this contingency. However, these tables present a special feature: they do not distinguish between sexes, and this is a problem if the aim is to obtain results that distinguish between men and women. We therefore proceed to obtain an estimate of the probability of death at each age *x*, $q_{x}$, for men and another for women. This is done as follows:

- as the probabilities of death are known for each age by sex but not for the whole population at each age, the first step is to estimate what the probability of death is for each age from the information contained in the life tables for each sex. This was done by calculating a weighted average probability of death estimated for each age, ${\hat{q}}_{x}$, no matter what the sex of the individual. For this purpose, the set of $q_{x}$ from the life tables of the general population for each sex are used, that is, $q_{x}^{M}$ and $q_{x}^{F}$ (from now on, the superscripts *M* and *F* refer to male and female, respectively. As it was indicated in Section 3, the PERM/F-2000 tables are elaborated by sex but not for the whole population). In addition to these probabilities for each age, weighting factors are required. They are identified as ${\hat{\omega }}_{x}^{M}$ and ${\hat{\omega }}_{x}^{F}$. They are obtained from the structure figures of the general population in 2008 by age and sex, so that: ${\hat{\omega }}_{x}^{i}=\frac{P_{x}^{i}}{P_{x}}$ where $P_{x}^{i}$ is the total of the population with age *x* and belonging to sex *i* and $P_{x}$ is the total population with age *x*, whatever their sex were. So, the average is obtained as:
  (1) $${\hat{q}}_{x}=q_{x}^{M}{\hat{\omega }}_{x}^{M}+q_{x}^{F}{\hat{\omega }}_{x}^{F}$$
- the death probability for each sex at each age in general population, $q_{x}^{i}$ is then compared with the estimated average probability at that age. This provides a measure of the divergence between sex-specific and overall mortality for each age. This difference will be captured in a coefficient, called $rSS_{x}^{i}$, calculated as follows:
  (2) $$rSS_{x}^{i}=\frac{q_{x}^{i}}{{\hat{q}}_{x}}$$
  It must be said that the coefficients change with age and that there are two coefficients per each age.
- finally, once the former coefficients were calculated, the probabilities of death of the permanently disabled population are estimated for each sex as follows:
  (3) $${\hat{q}}_{x}^{SS_{i}}=q_{x}^{SS}rSS_{x}^{i}$$
- the estimated survival probabilities are obtained using this set of ${\hat{q}}_{x}^{SS_{i}}$ as ${\hat{p}}_{x}^{SS_{i}}=1-{\hat{q}}_{x}^{SS_{i}}$. Then, the values for $l_{x}^{SS_{i}}$ can be obtained as $l_{x}^{SS_{i}}=l_{x-1}^{SS_{i}}{\hat{p}}_{x-1}^{SS_{i}}$. The $l_{x}^{SS_{i}}$ profiles can be seen in Figure 1:

Step 2: Elaboration of life tables for each degree of dependency, sex and survival scenario.

In this stage, life tables are generated for the dependent population for each of the degrees recognized by the Spanish law. As it was pointed out in Section 1 there are papers suggesting that the residual lifespan for people in this group is shorter than that of the general population. However, there are no life tables for dependent persons in Spain. The most similar are the Social Security tables used in this paper. The problem with this table is that for each age it shows the same probability of death regardless of the intensity of dependency. For this reason, and to achieve greater detail in the calculation, different scenarios are considered. In all of them it is assumed that the probability of survival is lower in the dependent population than in the general population at the same age. The information necessary to construct these tables comes from EDAD 2008. It was proceeded to reconstruct the history of the dependency scale from the information on the age at which each of the disabilities was registered. From now on, the value of the individual dependency scale will be expressed by *B*. Besides this information, we have used the sex of each record, its elevation factor (number of persons that each record represents) and the age that the individual had at the time of carrying out the survey. Once the history of scales was generated for each individual, we proceed to calculate the average scales by age and grade considered (0, I, II, and III). The age range considered is that included in EDAD 2008. The maximum ages are 102 and 104 for men and women, respectively. Once the average scales in all cases are obtained, it is necessary to make a number of assumptions to estimate the reduction in the probability of survival in the dependent population relative to that of the general population, *r*. For this ratio, the following assumptions were made (A1 to A4):

- A1: this ratio is equal to $\frac{{\hat{p}}_{x}^{SS_{i}}}{p_{x}^{i}}$ when the scale *B* is equal to the average, i.e., $B=\bar{B}$. This case is denoted as $r_{\bar{B}}$.
- A2: this ratio tends to unity in the case where there is no dependence (B=0).
- A3: this ratio is assumed to be less than unity. In the absence of statistical information, 10 values are assumed, denoted henceforth as *k*, from 0.8 to 0.98, with steps of 0.02.
- A4: finally, the definitive reduction depends both on the scale for each degree and on age, so that the higher the scale, and therefore the more intense the dependency, the greater the reduction in the probability of survival.

The final graduation of the *r*-values and thus the estimated $q_{x}$ depend on the value of the individual’s scale in relation to the average scale. Two different situations are distinguished:

- $B>\bar{B}$. In this case, the two extreme situations occur when *B* values are $\bar{B}$ and 100. As it was previously indicated when the scale is equal to the average, then *r* is equal to $r_{\bar{B}}$. From this point, the ratio decreases as the scale increases until it becomes equal $kr_{\bar{B}}$ to when the scale reaches 100 points. Therefore, when the scale reaches that maximum point, the largest variation of the scale and then, $\Delta B=100-\bar{B}$, it follows that $\Delta r=kr_{\bar{B}}-r_{\bar{B}}$. So, for any change equal to $B-\bar{B}$, it follows that $\Delta r=\frac{\left(k-1\right)\left(B-\bar{B}\right)}{100-\bar{B}}r_{\bar{B}}$. According to this, the final ratio, $r_{f}$, is equal to $r_{\bar{(}B)}+\Delta r$, that is:
  (4) $$r_{f}=r_{\bar{B}}-\frac{\left(1-k\right)\left(B-\bar{B}\right)}{100-\bar{B}}r_{\bar{B}}.$$
- $B<\bar{B}$. In this case, the two extreme situations occur when B values are 0 and $\bar{B}$. In the first case there is no dependence. We call the ratio in this case $r_{0}$ and its definition is explained later. Therefore, as *B* increases from 0 to $\bar{B}$, the ratio goes from $r_{0}$ to $\bar{B}$. Therefore, for any value of *B* between these two extremes, changes in *r* is equal to $\frac{B}{\bar{B}}\left(r_{\bar{B}}-r_{0}\right)$, so that the final ratio, $r_{f}$, is equal to $r_{0}-\frac{B}{\bar{B}}\left(r_{\bar{B}}-r_{0}\right)$. The value of $r_{0}$ remains to be determined. This ratio is associated with a situation without dependence. This would make it equal to unity. However, to consider the different scenarios of intensity of this contingency, the effect of assuming different scenarios of intensity in the loss of survival in this group was subtracted from this unit value, so that the value taken for $r_{0}$ is $1-\left(1-r_{\bar{B}}\right)\left(1-k\right)$. The first bracket refers to the loss associated with suffering this contingency, whereas the second one reflects the intensity of the loss.

Once the final ratios of survival deterioration due to dependency were estimated, the $q_{x}$ sought are obtained as ${\tilde{q}}_{x}^{i}=1-r_{f}p_{x}^{SS_{i}}$. In this way, the estimated ${\tilde{q}}_{x}^{i}$ is obtained for the four degrees considered in each of the 10 scenarios considered, generating the life tables. Finally, the results are smoothed using GAM models based on cubic splines.

Step 3: Estimation of the transition matrices between all the states considered.

To calculate the transition probabilities, it is assumed that an improvement of the personal dependency situation is not possible, as suggested by [27] (stand-alone model without improvement). They are calculated using two sources of information. On the one hand, the transition probabilities from one state to another of the dependency are obtained from EDAD 2008. Thus, let $P_{x0}^{ij}$ be the probability that a person who is in state *i* at age *x* moves to state *j* at age x+1. It is calculated as the proportion of individuals who satisfy this condition in relation to all individuals with age x, whatever were their situation. These probabilities are calculated without considering death. These probabilities are calculated from the information contained in EDAD-2008. Therefore, it is possible that the probabilities obtained were affected by the data selected in the sample, and they may not adequately reflect the dynamics of the ages analyzed. To include this state, it is necessary to use the life tables obtained in Step 2. It is obtained the following probabilities:

- the final probability of passing from state *i* to *j* at age *x* and still being alive at x+1, $P_{xf}^{ij}$, is equal to $P_{x0}^{ij}P\left(\bar{D}\right)\;\forall i,j$ where $P(\bar{D})$ refers to the probability of being alive at age *x*.
- the final probability of passing from state *i* at age *x* to be dead at age x+1, $P_{xf}^{iD}$ is obtained as $1-\sum_{i\leq j}^{4}P_{xf}^{ij}$
- as there is no improvement in the state of the dependency suffered then $P_{xf}^{ij}=0\;\forall i>j$.
- finally and obviously, $P_{xf}^{DD}=1$.

This gives the transition matrices from age *x* to age x+1 for the scenario *k* (henceforth, D(x,x+1,k).

Step 4: Derivation of life expectancies for each age, sex, degree, and scenario.

Since the majority of dependents are at least 50 years old, the calculation is made from this age onwards. The following procedure will be followed:

- firstly, the matrix is obtained for the age of 50 years old, and a given scenario *k*, D(50,51,k) is taken as the origin of the process (k=1,…,10)
- assuming that the transition matrices for each age are reflecting Markov homogeneous chains, then:
  (5) $$D\left(x,t,k\right)=\prod_{z=x}^{t-1}D\left(z,z+1,k\right)\;50\leq x<t\leq \omega$$
  These are the matrices used to estimate the ${}_{t}p_{x}$ needed to obtain the life expectancies.
- for each age, degree, sex, and scenario, the ${}_{t}p_{x}$ are obtained as the horizontal sum in the matrices D(x,t,k) of the probabilities of passing from a certain state to a state other than death.
- finally, the life expectancies at a certain degree, age, scenario, and sex are obtained as:
  (6) $$e_{x}\left(g,k,s\right)=\frac{1}{2}+\sum_{x=z}^{\omega }{}_{t}p_{x}\left(g,k,s\right)$$

## 5. Results

**Figure 2:**
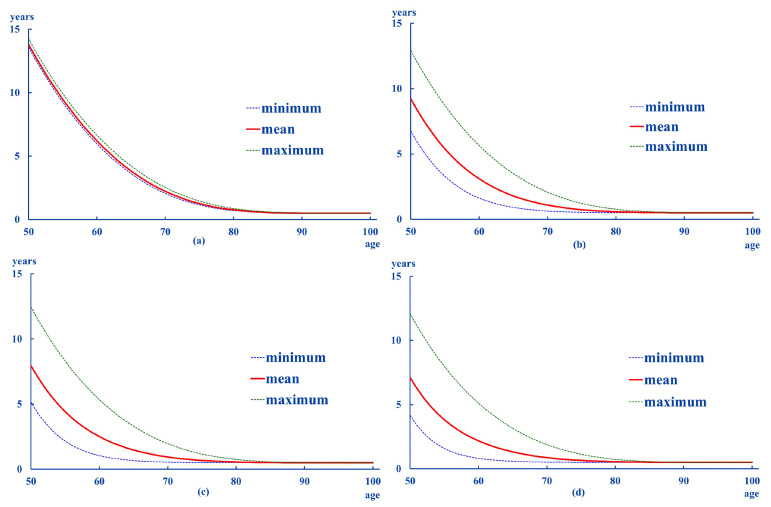
$e_{x}$: men. (**a**): Degree 0, (**b**): Degree I, (**c**): Degree II, (**d**): Degree III.

**Figure 3:**
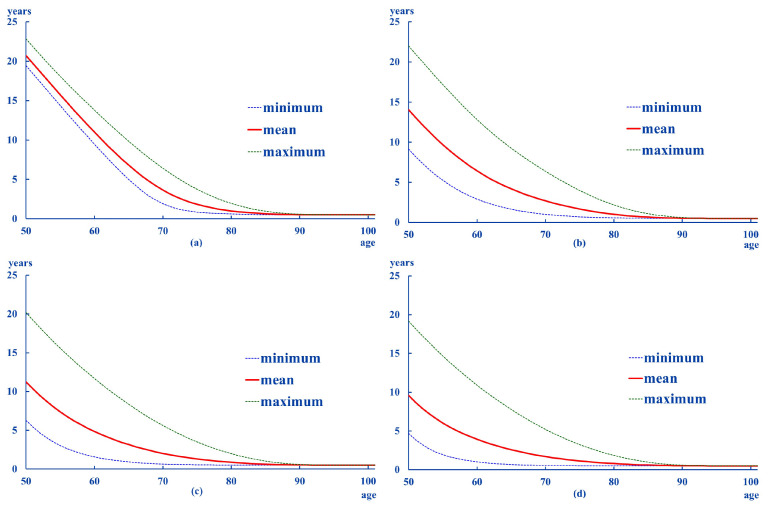
$e_{x}$: women. (**a**): Degree 0, (**b**): Degree I, (**c**): Degree II, (**d**): Degree III.

The life expectancies obtained in the previous section suggest different patterns between men and women. Thus, Figure 2 and Figure 3 show the evolution of life expectancies for each of the sexes for ages between 50 and 100 years old. As they were estimated for each of the scenarios considered, the graphs only show the average, the maximum and minimum values.

The values for each age are higher for women than for men. Table 1 and Table 2 summarize the pattern of life expectancy by both sex and degree:

Once life expectancies were calculated, it may be interesting to determine how many years an individual starting at a certain age in a certain degree is expected to spend in each of the degrees under consideration. To do so, the transition probabilities between the ages to be analyzed and the final age considered is used. Table 3 and Table 4 show the transition probabilities at the ages 50, 60, 70, and 80 for men and women, respectively:

From the information contained in the above tables, the following can be deduced:

- average life expectancies are higher in women than in men, as it happens in the general population.
- except for Degree 0, the age at which the $e_{x}$ is less than one year of life is 8 to 10 years higher in women than in men. This difference becomes smaller as the intensity of dependency increases.
- the distances from the mean to the extremes are larger for women than for men. This pattern is also registered in the standard deviations of these differences.
- in general, the results suggest greater and more dispersed distances in the high ranges (maximum–average).
- low degrees (0 and 1) show a relative stability in the transition probabilities, whatever the age considered. As for the probabilities associated with Degree II, there is a transfer to Degree III. This pattern can be seen in all the ages analyzed in women, whereas it can be seen from the age of 60 onwards in men.
- according to these results, it is most likely to remain at the Degree they were at the initial age. This pattern is more likely in men beginning in Degrees I and II. However, if their situation worsens, it is most likely to end at Degree III. This is more likely in women beginning in Degree II.

**Figure 4:**
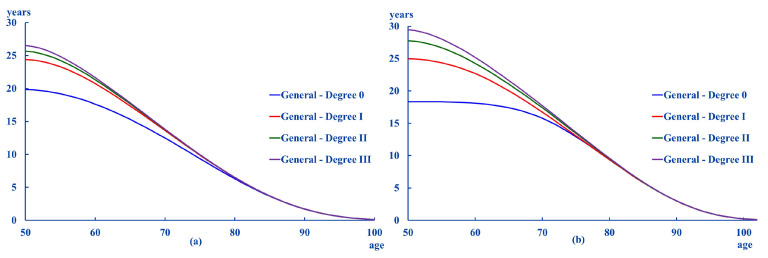
Differences in $e_{x}$ between general and dependent population. (**a**): men, (**b**): women.

Finally, Figure 4 shows the comparison between the $e_{x}$ for the general population and the dependent population by gender and grade:

Except for Degree 0, the differences between the two population groups are greater for women than for men. The figure suggests that there is an age beyond which this difference between the $e_{x}$ of the two populations does not depend on the intensity of dependency. This age is around 80 years for men and slightly earlier for women.

## 6. Discussion

Attention to the dependent population implies knowing not only what cares these people need, but also for how long. For this reason, it is appropriate to ask how many years would be the residual life expectancy of the individuals affected by this contingency.

This work does not pretend to know which part of a person’s residual life will be affected by dependency and which will not. The study assumes that all the individuals analyzed are already dependent. The aim of this work is different. Life expectancy was assessed at the level of an entire country. We took 50 years old as the starting age for the calculation, given that the vast majority of Spanish dependents are at least that age. In addition, a distinction was made by level of dependency, in accordance with Spanish legislation. Both institutionalized and noninstitutionalized were taken into account. Most Spanish dependents live at home, hence the group of institutionalized persons is less than 5% of the total. To this end, a multistate model was used, and five possible states were considered (the four associated with dependency according to Spanish legislation plus death). Based on the available statistical information and the established assumptions, life tables were prepared for each of the degrees considered (0, I, II, and III). Our contribution was the estimation of life expectancy, for which we have considered the transference between degrees over time, provided that this implies a worsening of the situation (stand-alone model). Therefore, the transition matrices between the states considered were estimated for all ages from 50 onwards. We also assume that for each age, the mortality of dependents will be higher than that of the general population. Since there is no statistical information on this aspect, 10 different scenarios were used. In each scenario, the probability of survival of a dependent is a fraction of the corresponding probability for an individual of the same age in the general population. As there is no statistical information about the differences in mortality between general and dependent population, it was assumed that all the 10 scenarios have the same probability of occurrence. The final estimated life expectancy was an average of the results obtained with each of the 10 scenarios considered.

The most relevant results obtained are shown next. Firstly, life expectancy for ages over 50 years old is higher in women than in men. Secondly, the age at which life expectancy is less than one year is 8 to 10 years higher in women than in men. Additionally, this difference narrows as the intensity of dependency increases. Thirdly, when comparing the life expectancy of the dependent population with that of the general population, it was found that the differences between the two groups are greater in women than in men. Besides, there is an age beyond which the difference between the life expectancy of both groups does not depend on the level of dependency. This age would be around 80 years in the case of men and slightly less in the case of women. Finally, the results suggest two patterns in the composition of life expectancy. The first one is that the most likely evolution is that people remains at the same Degree they were classified at the age of 50. The second pattern suggests that if the personal situation worsens, the most likely situation is becoming a dependent individual in Degree III.

Our results are not comparable with those obtained in previous studies reported in the Introduction. Most of them have estimated life expectancy in small and very localized groups, as [17] do. Others are specifically focused on studying the life expectancy of the population in nursing homes, such as [18,19] or [20]. Our results are also not comparable with [29] because they use a multistate model to estimate the number of years an individual remains in one of the states considered (healthy or unhealthy) before reaching the final state of death. Finally, our results are also not comparable with [28] because, although a large sample is used, the individuals studied are at least 65 years old. Furthermore, the aim of these authors is to estimate the probabilities of moving to and from long-term institutional care and probabilities of death and life expectancy in the community and in care by gender and marital status. The results obtained in this study are conditioned not only by the data used, but also by the definition of a dependent person and the different degrees of dependency contemplated in current Spanish legislation.

The results obtained open the way to different lines of future research. It is possible to refine the estimation of the transition probabilities between the different states by using parametric models. Another possibility is to consider that the reduction of the survival probabilities would not be uniform across the age structure. This means that the parameter *k* will be linked to age, that is, k(x). It is also possible to progress in the study of the economic consequences of dependence. In this sense, it would be possible to study the estimation of the individual cost of caring for dependents. Linked to this, it is possible to apply these costs in determining the premium for a long-term care insurance. This would be an improvement on the results shown in [36] where the premiums were obtained after assuming certain values for the care costs of these individuals. Finally, as life expectancy in western countries is increasing with the aging of the population, it would be desirable to study the evolution of life expectancy and its changes between degrees in the population aged 80 and over. It is expected that this group of individuals will represent the greatest social and economic costs in the next decades.

## Figures and Tables

**Table 1:** Summary of results (men).

			Mean-Minimum		Maximum-Mean	
	Mean	x for e < 1	Mean	Standard Deviation	Mean	Standard Deviation
Degree 0	3.29	77	0.11	0.10	0.21	0.18
Degree I	1.93	70	0.64	0.82	1.11	1.28
Degree II	1.65	69	0.66	0.89	1.25	1.51
Degree III	1.48	68	0.64	0.90	1.31	1.62

*Source*: own elaboration.

**Table 2:** Summary of results (women).

			Mean-Minimum		Maximum-Mean	
	Mean	x for e < 1	Mean	Standard Deviation	Mean	Standard Deviation
Degree 0	5.02	79	0.81	0.72	1.43	1.19
Degree I	3.33	80	1.55	1.70	2.91	2.88
Degree II	2.60	78	1.43	1.66	3.05	3.15
Degree III	2.19	76	1.30	1.57	3.12	3.31

*Source*: own elaboration.

**Table 3:** Transition probabilities starting at certain ages (men).

	*x* = 50					*x* = 60			
	D 0	D I	D II	D III		D 0	D I	D II	D III
D 0	0.56	0.00	0.14	0.29	D 0	0.56	0.00	0.15	0.29
D I	0.00	0.56	0.13	0.31	D I	0.00	0.57	0.12	0.31
D II	0.00	0.00	0.78	0.22	D II	0.00	0.00	0.80	0.20
D III	0.00	0.00	0.00	1.00	D III	0.00	0.00	0.00	1.00
	***x* = 70**					***x* = 80**			
	D 0	D I	D II	D III		D 0	D I	D II	D III
D 0	0.55	0.01	0.15	0.30	D 0	0.52	0.02	0.15	0.31
D I	0.00	0.53	0.14	0.33	D I	0.00	0.52	0.14	0.34
D II	0.00	0.00	0.71	0.29	D II	0.00	0.00	0.69	0.31
D III	0.00	0.00	0.00	1.00	D III	0.00	0.00	0.00	1.00

*Note*: D means Degree. *Source*: own elaboration.

**Table 4:** Transition probabilities starting at certain ages (women).

	*x* = 50					*x* = 60			
	**D 0**	**D I**	**D II**	**D III**		**D 0**	**D I**	**D II**	**D III**
D 0	0.68	0.04	0.12	0.16	D 0	0.67	0.04	0.12	0.16
D I	0.00	0.48	0.18	0.34	D I	0.00	0.49	0.19	0.33
D II	0.00	0.00	0.60	0.40	D II	0.00	0.00	0.59	0.41
D III	0.00	0.00	0.00	1.00	D III	0.00	0.00	0.00	1.00
	***x* = 70**					***x* = 80**			
	**D 0**	**D I**	**D II**	**D III**		**D 0**	**D I**	**D II**	**D III**
D 0	0.66	0.05	0.12	0.17	D 0	0.63	0.05	0.12	0.19
D I	0.00	0.47	0.18	0.35	D I	0.00	0.43	0.18	0.39
D II	0.00	0.00	0.58	0.42	D II	0.00	0.00	0.54	0.46
D III	0.00	0.00	0.00	1.00	D III	0.00	0.00	0.00	1.00

*Note*: D means Degree. *Source*: own elaboration.

## Data Availability

Data was extracted from EDAD-2008. This survey can be found in INE website https://www.ine.es (Accessed on 15 December 2020).
